# Comparison of the RECIST 1.0 and RECIST 1.1 in patients treated with targeted agents: a pooled analysis and review

**DOI:** 10.18632/oncotarget.7322

**Published:** 2016-02-11

**Authors:** Jung Han Kim

**Affiliations:** ^1^ Department of Internal Medicine, Kangnam Sacred-Heart Hospital, Hallym University Medical Center, Hallym University College of Medicine, Seoul 07441, Republic of Korea

**Keywords:** RECIST 1.0, RECIST 1.1, targeted agent, tumor response, target lesion

## Abstract

Patients treated with targeted agents were not included in the data warehouse when the RECIST 1.1 was revised in 2009. We conducted this pooled analysis to investigate the impact of the RECIST 1.1 on the assessment of tumor response in cancer patients treated with targeted agents. We surveyed MEDLINE, EMBASE and PubMed for articles with terms of the RECIST 1.0 or RECIST 1.1. We searched for all the references of relevant articles and reviews using the ‘related articles’ feature in the PubMed. There were six articles in the literature comparing the clinical impacts of the RECIST 1.0 and RECIST 1.1 in patients treated with targeted agents for advanced or metastatic cancer. A total of 322 patients were recruited from the six trials; 217 with non-small cell lung cancer, 23 with thyroid cancer, 20 with gastrointestinal stromal tumor, and 62 with renal cell carcinoma. Because of new lymph node criteria, eight patients (2.5%) had no target lesions when adopting the RECIST 1.1. The number of target lesions by the RECIST 1.1 was significantly lower than that by the RECIST 1.0 (*P* < 0.001). However, the RECIST 1.1 showed high concordance with the RECIST 1.0 in the assessment of best tumor responses (*k* = 0.908). Seventeen patients (5.6%) showed discrepancy in the best tumor response between the RECIST 1.0 and RECIST 1.1. This pooled study demonstrates that the RECIST 1.1 shows the highly concordant response assessment with the RECIST 1.0 in patients treated with targeted agents.

## INTRODUCTION

The decision on subsequent cancer treatments usually depends on radiologic changes in the tumor burden. Thus, the accurate assessment of objective therapeutic response is essential for routine anti-cancer treatment as well as clinical trials using new drugs. Since the early 1980s, the World Health Organization (WHO) criteria were used as the standard method for evaluating tumor response [1]. According to the WHO criteria, tumors are measured bi-dimensionally by the product of the longest diameter and its longest perpendicular diameter for each tumor and tumor responses are classified into four categories by percentage changes in the sum of tumor measurements from baseline. Because the methods for selecting and measuring target lesions were not clearly described in the WHO guidelines, however, the assessment of tumor responses have been poorly reproducible between investigators [2, 3].

In 2000, the Response Evaluation Criteria in Solid Tumors (RECIST) Working Group proposed the new response evaluating criteria, the RECIST guidelines version 1.0 (RECIST 1.0) [4]. The important features of the RECIST 1.0 included the definition of minimum size of measurable lesions, the instruction on how many lesions to be assessed (up to 10, with a maximum of 5 per organ), and the use of uni-dimensional measurements, instead of the bi-dimensional criterion in the WHO guidelines. With an expectation of improving feasibility, the RECIST 1.0 had been widely accepted as the standardized method for tumor response assessment, especially in clinical trials with objective response or time to progression as primary end points. However, a number of questions and issues concerning the number of target lesions and the size of lymph nodes (LNs) to be measured have been raised with regard to the RECIST 1.0. In addition, recent rapid innovation of imaging technologies, such as multi-detector computed tomography (MDCT) and (^18^F) fluorodeoxyglucose-positron emission tomography (FDG-PET), requested an update of the RECIST 1.0 [5].

In 2009, the revised RECIST guidelines version 1.1 (RECIST 1.1) was presented to overcome the limitations of the original RECIST guidelines [6]. The RECIST 1.1 was based partly on the analyses of the database of about 6,500 patients with more than 18,000 target lesions from 16 clinical trials [7–9]. The major changes in the updated RECIST 1.1 include the reduction in the number of target lesions to be assessed (from 10 to 5 in total and from 5 to 2 per organ), the more stringent criteria for LN measurement, the augmented definition of disease progression, the new criteria for selecting bone lesions and cysts as target lesions, and the inclusion of FDG-PET in the detection of new lesions [6, 10–12]. Thereafter, the RECIST 1.1 has shown almost perfect agreement with the RECIST 1.0 in the assessment of tumor responses in patients receiving cytotoxic chemotherapy for advanced or metastatic non-small cell lung cancer (NSCLC) [13], gastric cancer (GC) [14, 15], and colorectal cancer (CRC) [16].

When the RECIST 1.1 was revised, patients treated with targeted agents were not included in the data warehouse [7]. Since the RECIST 1.1 was presented, a large number of agents targeting signaling pathway have been incorporated in the treatment of a variety of cancers. They are novel cytostatic biological agents acting as signal transduction inhibitors. With the increasing use of novel targeted agents, the RECIST 1.1, which was developed primarily for cytotoxic agents, needs to be verified if it can still applicable in patients receiving those agents. The RECIST 1.1 has shown a high concordance in several retrospective studies of patients treated with targeted agents [18–23]. However, each study had a small number of patients with a single type of primary cancer, so it is still necessary to evaluate how the RECIST 1.1 affects the assessment of tumor responses in patients treated with targeted agents. We conducted this pooled analysis to investigate the impact of the RECIST 1.1 on the selection of target lesions and classification of tumor response in patients treated with targeted agents for advanced or metastatic cancer, in comparison with the RECIST 1.0.

## RESULTS

### Eligible studies

There were eleven articles [13–16, 18–23] and one abstract [24] in the literature comparing the RECIST 1.0 and RECIST 1.1 in patients with solid tumors. However, four articles [13–16] compared the two criteria mainly in patients who had received cytotoxic chemotherapy and the abstract [24] focused on the measurement of the LNs, with little information about the tumor response. Finally, six studies [18–23] with patients treated with tyrosine kinase inhibitors (TKIs) for advanced or metastatic cancer were selected. There were no reports of patients treated with monoclonal antibodies.

### Patients' characteristics

A total of 322 patients treated with targeted agents for metastatic cancer were collected from the six trials; 217 with NSCLC [18–20], 23 with thyroid cancer (TC) [21], 20 with gastrointestinal tumor (GIST) [22], and 62 with renal cell carcinoma (RCC) [23]. The clinical characteristics of the patients were summarized in Table 1. However, two trials by Sun *et al.* [18] and Nishino *et al.* [19] had limited clinical information about the study patients. Almost all patients (96.9%) had at least one target lesion according to the RECIST 1.0. However, 8 patients (2.6%) had no target lesions when the RECIST 1.1 was adopted.

**Table 1:** Summary of the 6 studies comparing the RECIST 1.0 and RECIST 1.1

Characteristics	Sun *et al*. [18] NSCLC (*n* = 104)	Nishino *et al*. [19] NSCLC (*n* = 43)	Nishino *et al*. [20] NSCLC (*n* = 70)	Ruan*et al*. [21] TC (*n* = 23)	Shinagare *et al*. [22] GIST (*n* = 20)	Krajewski *et al*. [23] RCC (*n* = 62)
	no. of pts	no. of pts	no. of pts	no. of pts	no. of pts	no. of pts
Age, years	na	na	median 62	mean 54	mean 55	mean 61
(range)	-	-	(35–84)	(33–75)	(26–69)	(32–84)
Gender	na	na				
Male	-	-	12 (17.1%)	14 (60.9%)	14 (70%)	44 (71%)
Female	-	-	58 (82.9%)	9 (39.1%)	6 (30%)	18 (29%)
Histology	na	na				
Adenocarcinoma	-	-	63 (90%)	-	-	-
Well/moderately differentiated	-	-	na	-	-	-
Poorly differentiated	-	-	na	-	-	-
Non-adneocarcinoma	-	-	7 (10%)	-	-	-
GIST	-	-	-	-	20 (100%)	-
Follicular	-	-	-	1 (4.4%)		-
Papillary	-	-	-	22 (95.6%)	-	21 (33.9%)
Clear cell	-	-	-	-	-	38 (61.3%)
Chromophobe	-	-	-	-	-	2 (3%)
Others*	-	-	-	-	-	1 (1.6%)
Target lesions by RECIST 1.0	104 (100%)	43 (100%)	69 (98.6%)	14 (60.9%)	20 (100%)	62 (100%)
Median target lesions†(range)	0	2 (1–9)	2 (0–10)	3 (1–6)	na	4 (1–10)
No target lesion by RECIST 1.1	0	3 (6.9%)	2 (2.9%)	0	0	3 (4.8%)
PET	na	6 (4.3%)	10 (14.3%)	5 (21.7%)	na	very few
Treatment						
Erlotinib	36 (34.6%)	43 (100%)	63 (90%)	0	0	0
Gefitinib	68 (65.4%)	0	7 (10%)	0	0	0
Sorafenib	0	0	0	23 (100%)	0	na
Regorafenib	0	0	0	0	20 (100%)	0
VEGFR-TKI^‡^	0	0	0	0	0	62 (100%)

Abbreviations: NSCLC, non-small cell lung cancer; GC, gastric cancer; CRC, colorectal cancer; RCC, Renal cell carcinoma; TC, thyroid cancer; na, not available; no. of pts, number of patients; PET, positron emission tomography; VEGF, vascular endothelial growth factor receptor tyrosine kinase inhibitors.

* predominant sarcomatoid features with clear cell histology.

† according to the RECIST 1.0.

‡ includes tivozanib, pazopanib, foretinib, sorafenib, vatalanib, and sunitinib.

Patients with metastatic NSCLC were treated with epidermal growth factor receptor TKIs (EGFR-TKIs) such as gefitinib and elrotinib [18–20]. All patients with radioactive iodine-refractory TC received sorafenib, a multi-target, small molecule TKI [21]. Patients with GIST were all treated with regorafenib after failure of imatinib and sunitinib [22]. Patients with RCC received one of the vascular endothelial growth factor receptor TKIs (VEGFR-TKIs) such as tivozanib, pazopanib, foretinib, sorafenib, vatalanib, or sunitinib [23].

### Number of target lesions

The data about the number of target lesions was available in four studies [19–21, 23]. The number of target lesions according to the RECIST 1.1 was significantly lower than that according to the RECIST 1.0 (*P* < 0.001, paired Student's *t*-test). The median number of target lesions was 3 (range, 1–10) by the RECIST 1.0 and 2 (range, 0–5) by the RECIST 1.1, respectively. Among 188 patients who had at least one target lesion from the 4 studies, 104 (55.3%) showed a decrease in the number of target lesions when the RECIST 1.1 was used (Table 2). The new LN criteria of the RECIST 1.1 (LNs should be more than 15 mm in the short axis to be considered pathological) led to the reduction of target lesions in 53 patients (28.2%). In 37 patients (19.7%), the decreased number of target lesions was resulted from the reduction of the maximum number of target lesions in the RECIST 1.1 (up to five in total and up to two per organ). Eighteen patients (9.6%) showed a decrease in the number of target lesions because of both the LN criteria and the reduction of maximum target lesions. Out of 312 patients who had target lesions according to the RECIST 1.0, 8 (2.6%) had no target lesions when adopting the RECIST 1.1. In these patients, all the target lesions according to the RECIST 1.10 were LNs smaller than 15 mm along the short axis and no longer met the RECIST 1.1 criteria for target lesions.

**Table 2:** Decrease in the number of target lesions by adopting the RECIST 1.1

Reference	Tumor type	No. of patients			
		Loss of target lesion	Reduction of target lesions	Causes of reduction	No. of patients
Nishino *et al.* [19]	NSCLC	3	22	LN criteria Maximum no. of target lesions	18 4
Nishino *et al.* [20]	NSCLC	2	31	LN criteria Maximum no. of target lesions Both	24 1 6
Ruan *et al.* [21]	TC	0	8	LN criteria Maximum no. of target lesions Both	1 5 2
Krajewski *et al.* [23]	RCC	3	43	LN criteria Maximum no. of target lesions Both	10 27 6

### Tumor responses

We compared the best tumor responses between the two criteria in 304 patients who had at least one target lesion by the RECIST 1.1 (Table 3). The remaining 18 patients were excluded from the analysis because they had no target lesions according to the RECIST 1.1 and their tumor responses were unknown. The best tumor responses between the RECIST 1.0 and RECIST were highly concordant in patients treated with targeted agents (linear weighted *k* = 0.908, 95% confidence interval, 0.872–0.945). The ORRs, which were estimated in total regardless of the primary tumor site, were not significantly different between the two criteria (41.1% by the RECIST 1.1 versus 36.2% by the RECIST 1.0, *P* = 0.212).

**Table 3:** Comparison of the tumor responses by the RECIST 1.0 versus RECIST 1.1

Tumor response by RECIST 1.0	Tumor response by RECIST 1.1				Total
	CR	PR	SD	PD	
CR	1	0	0	0	1
PR	2	107	0	0	109
SD	0	15	111	4	130
PD	0	0	2	62	64
Total	3	122	113	66	304

Abbreviations: CR, complete response; PR, partial response; SD, stable disease; PD, progressive disease.

The level of concordance of tumor responses between the RECISI 1.1 and RECIST 1.0 is 0.908 (liner weighted k, 95% CI 0.872–0.945).

The overall response rates were not significantly different between the two criteria (41.1% by the RECIST 1.1 versus 36.2% by the RECIST 1.0, *P* = 0.212).

Seventeen patients (5.6%) showed discrepancy in the assessment of tumor responses between the RECIST 1.0 and RECIST 1.1. The details of the patients showing disagreement between the two criteria was described in Table 4. The disagreement of the best tumor responses between the two criteria were between partial response (PR) and stable disease (SD) in 9 patients, SD and progressive disease (PD) in 6, and PR and CR in 2. No patients showed discrepancy between PR and PD. When adopting the RECIST 1.1, the best tumor responses were upgraded in 13 (76.5%) of 17 patients with the disagreement between the two criteria: from PR to complete response (CR) in 2, from SD to PR in 9, and from PD to SD in 2. The major cause of disagreement in the best tumor response was the new LN criteria, which led to the different response classification in 8 patients (47.0%). Two NSCLC patients with SD according to the RECIST 1.0 were defined as PD because of the new lesions noted on PET scans.

**Table 4:** Summary of the patients showing disagreement between the RECIST 1.0 and RECIST 1.1

Reference	Tumor type	Tumor response		No. of patients	Causes of disagreement
		RECIST 1.0	RECIST 1.1		
Sun *et al*. [18]	NSCLC	PR	CR	2	LNs < 10 mm
		SD	PR	3	Equivocal LNs
		SD	PD	1	A definitely increased LN
Nishino *et al*. [19]	NSCLC	SD	PD	2	New lesions on PET
		PD	SD	1	A single LN < 10 mm
Nishino *et al*. [20]	NSCLC	SD	PR	1	Decreased number of target lesion
Ruan *et al*. [21]	TC	SD	PR	1	Not described
Krajewski *et al*. [23]	RCC	PD	SD	1	LN < 15 mm and up to 2 target lesion per
		SD	PD	1	organ
		SD	PR	4	Three LNs targets by the RECIST 1.0 Not described

## DISCUSSION

The RECIST 1.1 has shown high concordance with the RECIST 1.0 in the assessment of tumor responses in several studies with a small number of patients treated with targeted agents [18–23]. In this pooled analysis using those studies, we investigated the impact of the RECIST 1.1 on the selection of target lesions and the assessment of tumor response in patients who had been treated with targeted agents. Although the RECIST 1.1 significantly decreased the number of target lesions to be measured in those patients, there was an excellent agreement in the assessment of the best tumor response between the RECIST 1.0 and RECIST 1.1.

As expected, the RECIST 1.1 significantly decreased the number of target lesions. Among 188 patients who had at least one target lesion from the 4 studies in which the number of target lesions were described [19–21, 23], 157 (61.6%) showed a decrease in the number of target lesions when the RECIST 1.1 was used. The maximum number of target lesions to be assessed in the RECIST 1.1 was reduced from 10 to 5 in total, and from 5 to 2 per organ. The decreased maximum number of target lesions in the RECIST 1.1 resulted in the decreased number of target lesions in 51 patients (27.1%). The lytic or mixed lytic-blastic bone lesions with an identifiable soft tissue component may be regarded as target lesions according to the RECIST 1.1. In this pooled study with 322 patients, however, only one with TC was identified to newly have a bone target lesion when adopting the RECIST 1.1. The main cause of the reduction in the number of target lesions was different according to the primary tumor types. While the new LN criteria was the major case of the reduction of target lesions in patients with NSCLC, the reduction in the maximum number of target lesions was the dominant cause in patients with TC or RCC.

The RECIST 1.1 recommends that LNs should be measured along its short axis, regarding LNs of at least 15 mm as target lesions. LNs with at least 10 mm but less than 15 mm in its short axis, even though it may be pathological, are regarded as non-target lesion, and LNs with a short axis of less than 10 mm are recorded as normal. This new LN criteria were the most common cause of the reduction of target lesions in this pooled analysis, which led to the reduction of target lesions in 67 patients (35.6%), including 14 (7.4%) in whom the reduction was resulted from both the new LN criteria and the reduction in the maximum number of target lesions. In this pooled study with 312 patients who had target lesions according to the RECIST 1.0, 8 (2.6%) had no target lesions when adopting the RECIST 1.1. In these patients, all the target lesions according to the RECIST 1.0 were LNs smaller than 15 mm along the short axis, which was no longer met the RECIST 1.1 criteria for target lesions. Similar findings have been shown in patients with GC [15] or CRC [16]. Fuse *at al.* reported that 66 (38%) out of 172 LNs regarded as target lesions by the RECIST 1.0 were defined as target lesions based on the RECIST 1.1 in patients with metastatic GC [15]. In the study by Jang *et al.*, only 38 (40%) out of 95 LNs considered to be target lesions according to the RECIST 1.0 were classified as target lesions by the RECIST 1.1 in patients with metastatic CRC [16]. These findings indicate that the new LN criteria of the RECIST 1.1 may alter the eligibility of patients for clinical trials in which the ORR or time to progression is a primary endpoint.

When the updated RECIST 1.1 was presented in 2009, patients treated with targeted agents were not included in the data warehouse [7]. Recently we reported a pooled analysis of studies comparing the RECIST 1.0 and RECIST 1.1 in patients with metastatic cancer [25]. However, the data of the published paper also included patients treated with cytotoxic chemotherapeutic agents [14, 16]. In this pooled study only with patients treated with targeted agents, the RECIST 1.1 also showed high concordance with the RECIST 1.0 in the assessment of tumor responses. In 304 patients who had at least one target lesion according to the RECIST 1.1, the level of agreement in the best tumor responses between the two criteria was very high, with a linear weighted kappa value of 0.908. Seventeen patients (5.6%) showed the disagreement between the two criteria. The major cause of discordance in the best tumor response between the two criteria was the new LN criteria (8 patients). Because patients with either PR or SD stay on the same treatment in clinical practice, patients showing discordance between PR and SD would have no significant clinical impact. In this pooled study, only six patients (1.9%) revealed discrepancy between SD and PD. Therefore, the clinical impact of the RECIST 1.1 on changing therapeutic decisions seemed to be minimal. Regardless of the primary site, the estimated ORRs were not significantly different between the two criteria (41.1% by the RECIST 1.1 versus 36.2% by the RECIST 1.0, *P* = 0.212). Of note, however, the best tumor response tends to be upgraded in some patients when adopting the RECIST 1.1. Thirteen (76.5%) of 17 patients with the disagreement between the two criteria showed the better response classification according to the RECIST 1.1: from PR to CR in 2, from SD to PR in 9, and from PD to SD in 2. This finding may be mainly resulted from the more stringent LN criteria in the RECIST 1.1. In the study by Sun *et al.* [18], two NSCLC patients with PR by the RECIST 1.0 were re-classified as CR because LNs with short axes of < 10 mm were considered normal based on the RECIST 1.1.

The RECIST working group recently reported the results of a survey in oncology communities assessing satisfaction with the current RECIST 1.1, areas of concern, and suggestions for a new version of the RECIST [26]. Of 65 replies, 52.3% of responders were satisfied with the current RECIST 1.1 while 10.8% showed dissatisfaction. Area of weakness indicated in the RECIST 1.1 included the absence of potential early indicators of response such as functional imaging, the scarceness of validation in rare tumors, and the lack of validation for novel targeted agents. Targeted agents tend to induce necrosis and cystic change in solid tumors without necessarily producing tumor shrinkage [17]. Anatomic imaging alone may have limitations, particularly in assessing the activity of targeted therapies that stabilize diseases. The RECIST version 1.1 includes PET scans for the detection of new lesions. 18F-FDG PET is also increasingly adopted to monitor tumor responses to targeted therapies in solid tumor [27]. It has been shown to correlate well with anatomic response and, in some cases, even survival with targeted therapy in patients with solid tumors [28, 29]. The PET response criteria in solid tumors (PERCIST) may provide clinicians more accurate information of therapeutic response in earlier stage of treatment [30]. Therefore, attempts to optimize the RECIST criteria are still needed to accurately evaluate tumor responses to targeted agents.

This pooled study has several limitations needed to be noted. First, a single radiologic method, CT, was mainly used for tumor measurements and PET was not routinely performed in all six studies. New lesions detected on PET scans change the tumor response from PR or SD to PD according to the RECIST 1.1. In this study, the best tumor response in two NSCLC patients was changed from SD to PD by the new lesions on PET scans. Second, the best tumor responses between the two criteria were compared only in patients with target lesions based on the RECIST 1.1. According to the RECIST 1.1, patients with SD or PR based on target lesion response are classified as PD, only when substantial progression of non-target lesions is observed. Based on the RECIST 1.0, however, the increase in size of only one or a few non-target lesions was also regarded as PD, although target lesions were stable or responding. Therefore, if the studies had included patients with non-target lesion, the new criteria for non-target lesions would have affected the concordance between the RECIST 1.0 and RECIST 1.1. Third, the data in this study were quite heterogeneous with relatively small number of patients with different tumor types and different targeted agents used (EGFR–TKI or VEGF–TKI). Therefore, it is necessary to verify the results in studies with larger homogeneous patients' cohort. Fourth, monoclonal antibodies such as cetuximab, bevacizumab or trastuzumab are frequently used to treat a variety of cancers. However, this study only included patients treated with TKIs. Finally, as I mentioned above, four articles [18–21] included in this study were also used in the previous pooled analysis comparing the RECIST 1.0 and RECIST 1.1 in patients treated with cytotoxic agents or targeted agents [25].

In conclusion, this pooled analysis demonstrates that the RECIST 1.1 provides highly concordant response assessment with the RECIST 1.0 in patients treated with targeted agents for advanced or metastatic cancer. With the increasing use of novel targeted agents, however, the RECIST 1.1 still needs to be verified if it can also applicable in patients receiving those agents.

## MATERIALS AND METHODS

### Searching strategy

We thoroughly looked into all potentially eligible studies through the following searching strategy. We surveyed the Cochrane Central Register of Controlled Trials (CENTRAL, Issue 9 of 12, September 2015), MEDLINE (from 2009 to September 2015) and EMBASE (from 2009 to week 36, 2015) for articles including the following terms in their titles, abstracts, or keywords; ‘RECIST 1.0 or RECIST 1.1’, ‘target lesion’ and ‘tumor response’. In addition, we searched for all the references of relevant articles and reviews. We used the ‘related articles’ feature in the PubMed to identify the related articles. We also investigated all abstracts presented in the conferences of the American Society of Clinical Oncology and European Society for Medical Oncology held between 2009 and 2015.

### Statistical analyses

The statistical significance of changes in the number of target lesions between the RECIST 1.0 and RECIST 1.1 was assessed using the paired Student's *t* test. Chi-square test was used to compare the overall response rates (ORRs) between two groups. *P*-values less than 0.05 were considered significant. The level of concordance of the best tumor responses between the two criteria was calculated using linear weighted ĸappa statistics. Agreement between the two criteria was interpreted as poor (*k* < 0), slight (*k* = 0–0.20), fair (*k* = 0.21–0.40), moderate (*k* = 0.41–0.60), substantial (*k* = 0.61–0.80), and almost perfect (*k* > 0.80) [31].
